# Crosstalk between angiogenesis and immune regulation in the tumor microenvironment

**DOI:** 10.1007/s12272-022-01389-z

**Published:** 2022-06-27

**Authors:** Hei Jung Kim, Young Rae Ji, You Mie Lee

**Affiliations:** 1 Vessel-Organ Interaction Research Center, VOICE (MRC), Kyungpook National University, 80 Daehak-ro, Buk-gu, Daegu, 41566 Republic of Korea; 2 National Institute on Deafness and Other Communication Disorders, National Institutes of Health, Bethesda, USA; 3 Department of Molecular Pathophysiology, Research Institute of Pharmaceutical Sciences, College of Pharmacy, Kyungpook National University, 80 Daehak-ro, Buk-gu, Daegu, 41566 Republic of Korea

**Keywords:** Tumor microenvironment, Angiogenesis, Immune suppressive tumor, Tumor-associated macrophage, Treg cells

## Abstract

Cancer creates a complex tumor microenvironment (TME) composed of immune cells, stromal cells, blood vessels, and various other cellular and extracellular elements. It is essential for the development of anti-cancer combination therapies to understand and overcome this high heterogeneity and complexity as well as the dynamic interactions between them within the TME. Recent treatment strategies incorporating immune-checkpoint inhibitors and anti-angiogenic agents have brought many changes and advances in clinical cancer treatment. However, there are still challenges for immune suppressive tumors, which are characterized by a lack of T cell infiltration and treatment resistance. In this review, we will investigate the crosstalk between immunity and angiogenesis in the TME. In addition, we will look at strategies designed to enhance anti-cancer immunity, to convert “immune suppressive tumors” into “immune activating tumors,” and the mechanisms by which these strategies enhance effector immune cell infiltration.

## Introduction

Over the past few decades, cancer research has focused on mechanisms of tumor formation and progression, especially as directed by oncogenes. However, in addition to genetic alterations within the tumor itself, the environmental niche surrounding the tumor can also contribute to cancer progression by releasing factors that promote cancer development or facilitate immune evasion (Hanahan and Weinberg 2011). In fact, solid malignancies are composed not only of tumor cells, but also vascular endothelial cells (ECs), fibroblasts, innate and adaptive immune cells, and extracellular matrix (ECM) components. The cells and external factors (cytokines, chemokines, growth factors, etc.) surrounding a tumor constitute the tumor microenvironment (TME). Immune cells found within the TME are often “tumor-associated” and promote tumor growth by playing an immunosuppressive function; immune cells within the TME include tumor-associated macrophages (TAMs), myeloid-derived suppressor cells (MDSCs), regulatory T cells (Treg cells), versus immune cytotoxic CD8^+^ T cells, CD4^+^ T cells, and natural killer cells (NK cells) (Whiteside 2008; Hanahan and Weinberg 2011). In addition, oxygen concentration, nutrient availability (glucose, amino acids, fatty acids, etc.), metabolites, and pH within the TME also play a role in cancer progression (Labani-Motlagh et al. 2020). In general, the TME is characterized by immunosuppression and abnormal vascularity, which acts as a major obstacle to chemotherapy (Munn and Jain 2019; Labani-Motlagh et al. 2020). Until recently, it was difficult to confirm sustained anti-cancer efficacy in clinical practice using only treatments that directly target cancer cells. Therefore, the effect of crosstalk between angiogenesis factors and immune cells within the TME on tumorigenesis and therapeutic efficacy should be considered. By understanding the characteristics of the TME and targeting specific components, such as factors promoting angiogenesis, it is expected that cancer growth and metastasis will be further inhibited and a lasting therapeutic effect will be achieved.

## Tumor angiogenesis and hypoxia

Angiogenesis is the formation of new blood vessels. This process involves the migration, growth, and differentiation of ECs, which line the blood vessels. While dormant tumors are devoid of active blood vessel formation during early stages of formation, active angiogenesis induced by angiogenic factors secreted from cancer cells themselves often contributes to escape from the dominant tumor (Hanahan and Weinberg 2011). Blood vessels are quiescent when pro- and anti-angiogenic factors are balanced. However, when the “angiogenic switch” occurs, pro-angiogenic signaling is upregulated compared to anti-angiogenic signaling to induce active blood vessel formation thus supplying the tumor with oxygen and nutrients resulting in the stimulation of tumor growth. Tumor progression is steadily accompanied by angiogenesis (Hanahan and Weinberg 2011; Jain 2014; Fukumura et al. 2018).

### Major angiogenic regulators in hypoxia

Hypoxia, reduced tissue oxygen tension, is often found in many solid tumors as a result of rapidly growing tumor cells. In normal tissues oxygen tension is over 40 mmHg, whereas in tumors it ranges between 0 and 20 mmHg (McKeown 2014). Hypoxia is a critical stimulator of angiogenesis from neighboring blood vessels, thus obtaining additional oxygen and nutrients. Hypoxia-inducible factors (HIF-1, -2, -3) are key transcription factors used by cells to adapt hypoxic stress in the microenvironment (Krock et al. 2011). Here, two major hypoxic signaling pathways and their relationship to tumor-adjacent immune responses and angiogenesis are introduced. Angiogenesis is a major outcome of HIF signaling as a response to hypoxic stress. Vascular endothelial growth factor (VEGF) family members induced by HIF signaling play the most critical role in angiogenesis by binding to their receptors VEGFR1-2 and neuropilin (Carmeliet and Jain 2000). The angiopoietin (Ang-1, -2)/Tie-2 pathway is a signaling pathway independent of the VEGF pathway. Ang-1 is required for normal vascular development, whereas Ang-2 is mainly expressed in remodeling tissues and in hypoxic TME (Fagiani and Christofori 2013). Elevated levels of VEGF and Ang-2 are correlated with a worse prognosis in various types of tumors (Lin et al. 2009; Canadas et al. 2015).

### Characteristics of tumor vasculature and hypoxic TME

**Fig. 1:**
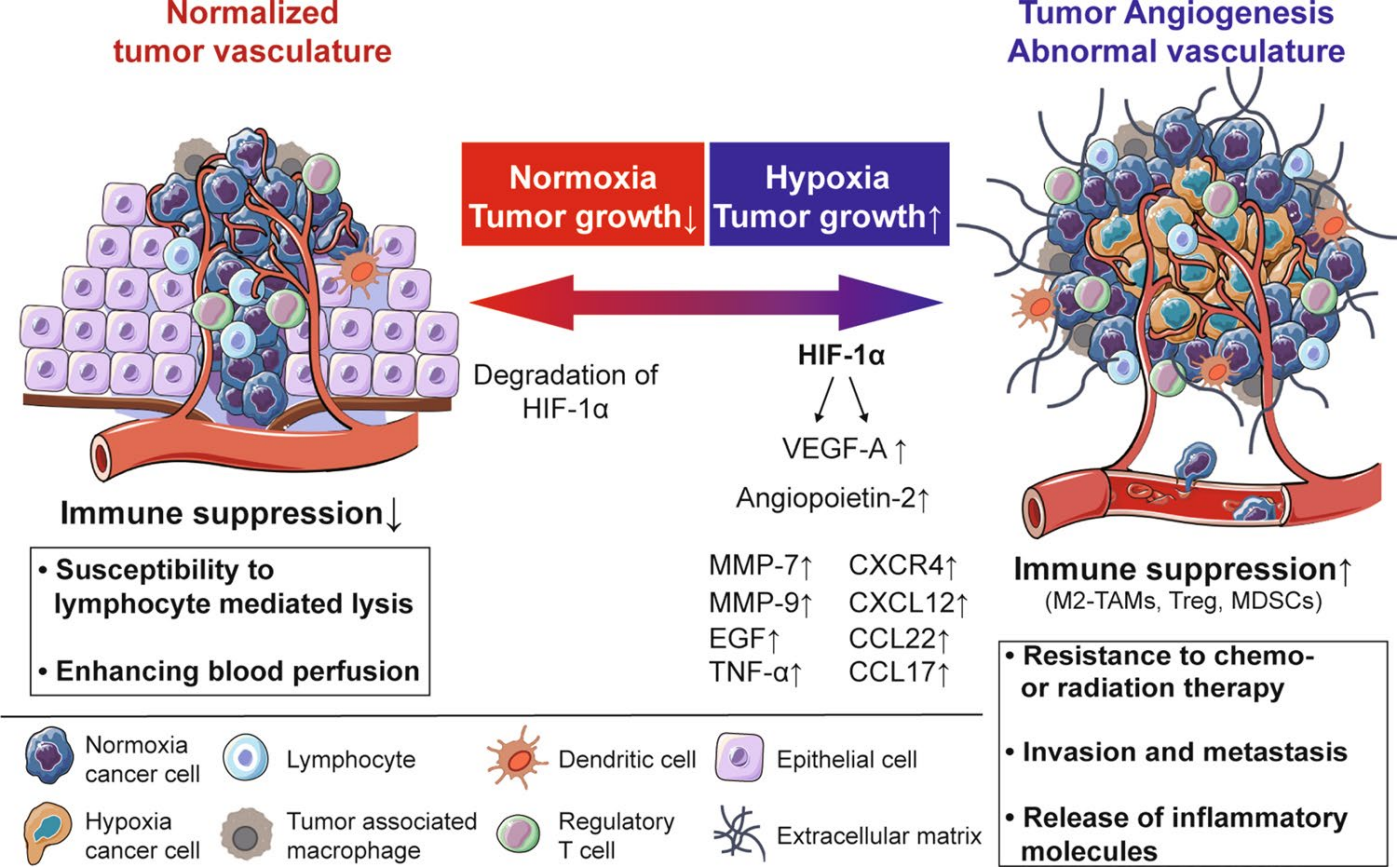
The environmental and metabolic pressures in the TME play key roles in tumorigenesis by impacting both the stromal and immune cell fractions, TME composition, and immune cell activation. Hypoxia promotes tumor angiogenesis and abnormal vascularization by activating HIF-1α, VEGF, and angiopoietin-2, which are associated with enhanced immune suppression, release of pro-inflammatory molecules, and promotion of tumor invasion and metastasis. The structural abnormalities of tumor vasculature increase the accumulation of regulatory T cells (Treg cells) and polarize tumor-associated macrophages (TAMs) to an immunosuppressive M2-like phenotype. The alleviation of tumor hypoxia through vascular normalization enhances blood perfusion, leading to the degradation of HIF-1α. The reestablishment of normal oxygenation additionally counteracts tumor growth through the revitalization of the anti-tumor immune response

Persistent high levels of angiogenic factors induce EC proliferation, migration, and venule formation in blood vessels. However, newly formed vascular networks may fail to mature and prune due to rapid formation compared to normal development. Vessel diameter is significantly uneven, poorly organized, and chaotic resulting in disrupted blood flow through malformed vessels. Tumor blood vessels tend to lose their normal barrier function via extensive branching and sprouting. This abnormality of tumor vasculature is caused by defects in pericyte coverage, resulting in leaking of interstitial fluids and blood cells (Jeong et al. 2021). This functional and structural abnormality of tumor blood vessels causes uneven blood flow within the tumor parenchyma, which can lead to areas of persistent or intermittent hypoxia (Kimura et al. 1996). Thus, despite being hyper-vascularized, the vascular network within the tumor tissue is often unable to supply oxygen or efficiently deliver anti-cancer drugs to the tumor cells due to lack of blood perfusion. Finally, hypoxic conditions within the solid tumor mass can induce expression of additional angiogenic factors, immune-checkpoint molecule PD-L1 (Noman et al. 2014), anti-apoptotic factors, and chemo- and radio-resistance molecules via HIF signaling/hypoxia (Semenza 2014; Xia et al. 2018) (Fig. 1; Table 1). Some of these molecules suppress the expression of adhesion molecules on tumor vascular endothelium, thus interfering with immune cell adhesion and migration across the vessel wall and preventing their infiltration into the tumor (Fukumura et al. 2018).

**Table 1:** Hypoxia-regulated genes involved in the regulation of the TME (Krock et al. 2011; Damgaci et al. 2018; Pietrobon and Marincola 2021)

Biological functions	Hypoxia/HIF target genes
Angiogenesis	VEGF, VEGFR-1, VEGFR-2, Angiopoietin-1, -2, Tie-2, ADM, FGF, PlGF, PDGF-B, SCF, Osteopontin, PAI-1, MMP, TIMP, NOS, COX-2, Endoglin, α1B-adrenergic receptor, Endothelin-1, Semaphorin 4D, Integrins, Leptin, Endosialin, Adenosine A_2A_ receptor, Oxygen-regulated protein-150, SDF-1, Interleukins (IL-1, IL-2, IL-4, IL-6, IL-8, IL-10, DLL, CTGF, HO-1)
Apoptosis	p53, BNIP-3, NIX, BAX, RTP801/RED1, Ref-1, Bcl-2, NF-kB, HSP70, BID
Proliferation/survival	IGF-BP1-3, IGF2, CCD1, TGF-α/β, p21, cyclin G2, NOS2
Cell migration and invasion	CXCR4, MMP-2, LOX, PAI-1, c-Met, LRP1, MIC2/CD99, fibronectin, uPAR, Col V, AMF/GPI, CATHD, integrin-linked kinase, integrins
Therapeutic resistance	MDR-1
Immune evasion	PD-L1, CD39, CD47, SDF-1 (CXCL12), ET-1, ET-2, FOXP3, CCL28, IFN-γ, IL-2, Semaphorin 3A

*ADM* adrenomedullin, *AMF* autocrine motility factor, *Ang-1/2* angiopoietin-1/2, *Bcl-2* B-cell leukemia/lymphoma 2, *BNIP3* Bcl-2 nineteen kilodalton interacting protein 3, *CATHD* cathepsin D, *CCD1* coiled-coil-DIX1, *CTGF* connective tissue growth factor, *COX-2* cyclooxygenase-2, *CXCR4* CXC chemokine receptor 4, *DLL* delta-like ligand, *HSP70* heat shock protein 70, *FGF* fibroblast growth factor, *HO-1* heme oxygenase-1, *IGF2* insulin-like growth factor 2, *IGF-BP* IGF factor binding protein, *LOX* lysyl oxidase, *MIC2* microneme protein 2, *MDR1* multidrug resistance 1, *MMP2* matrix metalloproteinase 2, *NF-κB* nuclear factor kappa B, *NOS2* nitric oxide synthase 2, *TGF-α*, *β* transforming growth factor-α, β, *uPAR* urokinase plasminogen activator receptor, *PAI-1* plasminogen activator inhibitor-1, *PD-L1*, *PlGF* placenta growth factor, *PDGF-B* platelet-derived growth factor beta, *SCF* stem cell factor, *SDF-1* stromal-derived growth factor, *Tie-2* TEK tyrosine kinase endothelial, *TIMP* tissue inhibitor of metalloproteinases, *VEGF* vascular endothelial growth factor, *VEGF-R* VEGF receptor

## Abnormal tumor vessels and immunosuppression

Abnormal blood vessels tend to exhibit high permeability, which increases interstitial fluid pressure but decreases blood perfusion, thereby restricting the entry of anti-cancer drugs and immune cells from the circulation into the cancer tissue. Abnormally leaky blood vessels facilitate intravasation, making it easier for cancer cells to migrate and metastasize to distant tissues (Jain 2014). Many factors, including VEGF in the TME, decrease the function of immune cells within the TME and prevent them from entering the tumor. In order for immune cells to enter the tumor from the blood vessels, the expression of adhesion molecules must be changed so that the immune cells can gather and adhere to the vascular ECs (Chen and Mellman 2013). However, angiogenic factors including VEGF change the expression of adhesion molecules such as intercellular adhesion molecule 1 (ICAM1) and vascular cell adhesion molecule 1 (VCAM1) in both EC and immune cells (Hendry et al. 2016). Moreover, substances such as CCL2, CCL28, CXCL8, CXCL12, Ang-2, VEGF, placental growth factor (PlGF), and adenosine secreted by cancer cells promote the recruitment of immunosuppressive cells, such as M2-TAMs, MDSCs, and Treg cells (Facciabene et al. 2011; Rolny et al. 2011; Chang et al. 2016). Then, various growth factors such as VEGF, Ang-2, and transforming growth factor-β (TGF-β) are produced and the recruited leukocytes further promote angiogenesis together with cancer cells and abnormal ECs to form an immunosuppressive state within the TME (Jain 2014) (Fig. 1).

In particular, VEGF has both a local and a systemic immunosuppressive function in cancer development. VEGF in the TME directly inhibits the trafficking, proliferation, and function of cytotoxic T lymphocytes (CTLs; Voron et al. 2015), resulting in an immunosuppressive effect. In addition, VEGF-VEGFR2 signaling inhibits antigen presenting by interfering with dendritic cells (DC) maturation, thus inhibiting T cell activity and resulting in decreased T cell-mediated anti-cancer activity (Gabrilovich et al. 1998). VEGF promotes the recruitment and proliferation of immunosuppressive cells such as Treg cells, MDSCs, and M2-TAMs; furthermore, excessive angiogenesis induced by VEGF creates a hypoxic TME following the formation of abnormal blood vessels, creating a more immunosuppressive environment (Huang et al. 2013).

The immunosuppressive function of Ang-2 is not well understood. However, it has been experimentally reported that Ang-2 signaling promotes immunosuppression. Ang-2 inhibits the binding of ECs and pericytes thus promoting the migration of immune cells from blood through ECs to the TME. Ang-2 also inhibits the secretion of TNF-α, thereby limiting the anti-cancer activity of monocytes (Fukumura et al. 2018). Ang-2 increases leukocyte–EC interactions by upregulation of adhesion molecules and promoting recruitment of MDSCs, Treg cells and Tie-2-expressing monocytes that induce immunosuppression (De Palma et al. 2005; Coffelt et al. 2011; Scholz et al. 2011). These reports suggest that abnormalities in cancer blood vessels are a critical factors influencing cancer progression, anti-cancer drug resistance acquisition, and patient prognosis via the inhibition of effector immune cell function within the tumor and the TME (Fig. 1).

## The contribution of immune cells in the tumor microenvironment

**Fig. 2:**
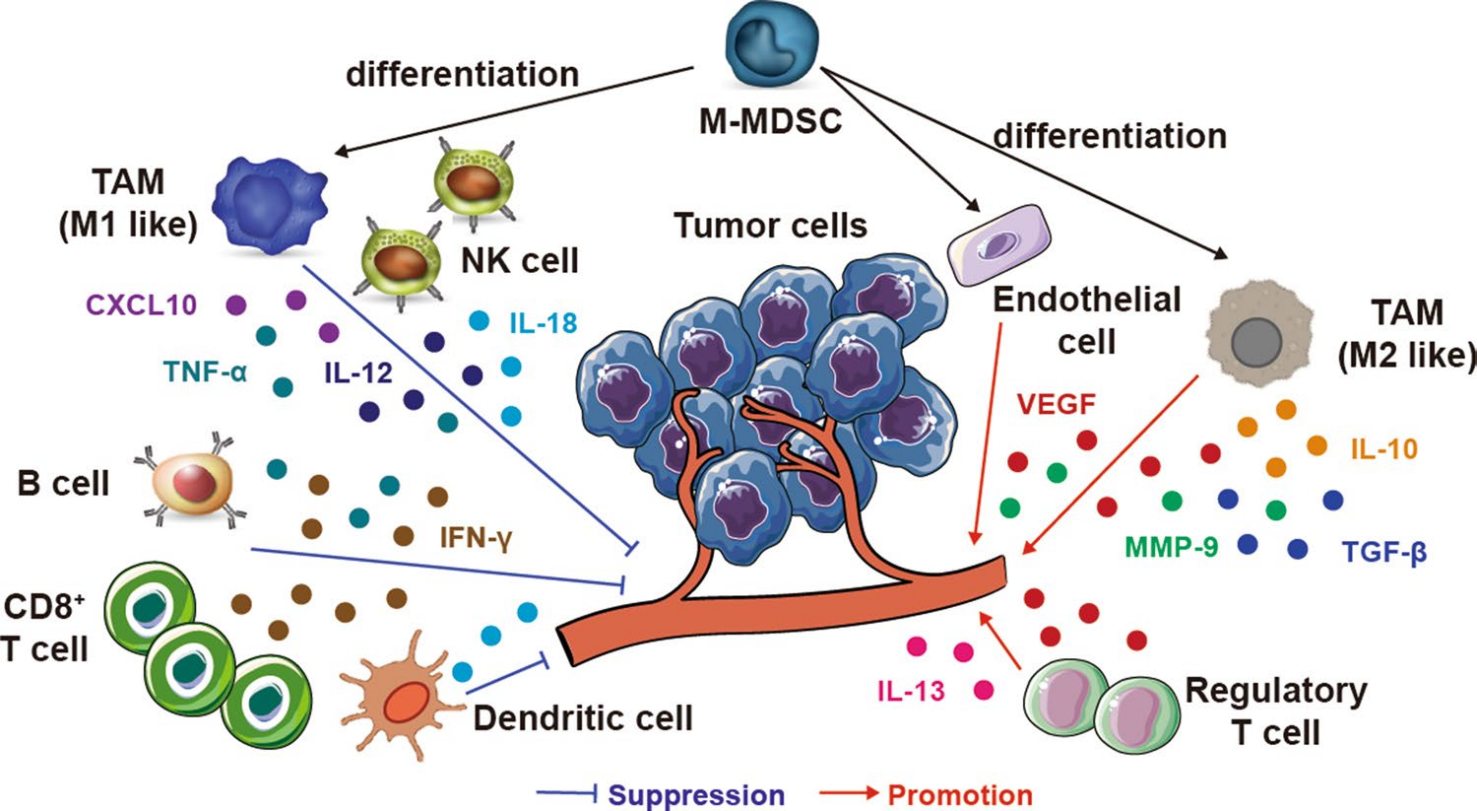
The main interactions between different immune cell types and the tumor vasculature in the tumor microenvironment. Immune cells directly influence the phenotypes and functions of tumor vessels through various cytokines. Innate immune cells, such as mature dendritic cells (mDCs) and M1-tumor-associated macrophages (TAMs), produce cytokines (IFN-α, IL-12, IL-18, or TNF-α) and chemokines (CXCL9, CXCL10, or CCL21) that suppress tumor angiogenesis. Meanwhile, adaptive immune cells, such as CD8^+^ T cells and T helper 1 (T_H_1) cells, secrete IFN-γ, a potent cytokine that inhibits angiogenesis and induces vascular normalization in the TME. However, myeloid-derived suppressor cells (MDSCs) and M2-TAMs significantly promote tumor angiogenesis by secreting VEGF, IL-10, Bv8, and MMP-9. Moreover, Treg cells can also release pro-angiogenic factors such as VEGF, IL-5, IL-13, and IL-17. In addition to direct effects on tumor vasculature, immune cells regulate tumor vasculature indirectly by communicating and polarizing with each other. mDC, CD8, and T_H_1 cells can skew macrophage polarization away from the M2 to the M1 phenotype. However, MDSCs and Treg cells can reprogram TAMs from M1 to M2

Immune cells within the TME play key roles against the development of cancer (Fig. 2). Immune cells influence the entire process of inflammation, immunity and tumorigenesis through direct or indirect mechanisms (Wang et al. 2019). Immune cells are involved in the production and release of numerous pro-vascular or anti-angiogenic factors, thereby regulating the formation of tumor vessels and the proliferation, migration, and activation of ECs (Shacter and Weitzman 2002; Conti et al. 2007; Disis 2010; Frenzel and Hermine 2013). In general, tumor-associated immune cells can be divided into tumor-suppressing and tumor-promoting immune cells based on their functions as summarized in Table 2.

**Table 2:** Tumor-associated immune cells within the TME and their related functions

Functions	Cell types	Role in tumor angiogenesis
Tumor-suppressing immune cells	Effector T cells	Killing of cancer cells by granule exocytosis and FasL-mediated apoptosis induction Polarizing M2-TAMs to M1-TAMs and induce DC maturation
	Effector B cells	Production of T_H_1 cytokines, enhanced CTL activity, and NK cell-meditated tumor cell killing
	Natural killer cells (NK cells)	Production of pro-inflammatory cytokines and chemokines, release of granules containing perforin and granzymes that induce the apoptosis of tumor cells
	Dendritic cells (DCs)	Processing and presentation tumor antigens to naïve T cells Production of IL-12 and IL-18 to induce the activation and proliferation of Treg cells
	M1-tumor-associated macrophages (TAMs)	Promotion tumor elimination through secretion of pro-inflammatory cytokines and generation of high levels of reactive oxygen/nitrogen species Induction vessel maturation by secreting anti-angiogenic cytokines
Tumor-promoting immune cells	M2-tumor-associated macrophages (TAMs)	Promotion of tumor escape by inducing anti-inflammatory T_H_2 responses through secretion of IL-10 and TGF-β
	Regulatory T cells (Treg cells)	Secreting cytokines such as IL-10, TGF-β; establishing an immunosuppressive environment; associated with poor prognosis
	Myeloid-derived suppressor cells (MDSCs)	Inhibition of T cell function by production of iNOS from arginine and immunosuppressive cytokines Associated with tumor progression and neo-angiogenesis; suppressing T cells and NK cells; differentiating into TAMs under hypoxic conditions
	Regulatory B cells (Breg cells)	Production of IL-10 and IL-35 to inhibit effector T cell function and promote an immunosuppressive environment

## Tumor-suppressing immune cells

The tumor-suppressing immune cells mainly consist of effector T cells (including cytotoxic CD8^+^ T cells and effector CD4^+^ T cells), B cells, NK cells, DCs, and M1-tumor associated macrophages.

### Effector T cells

In the TME, CD8^+^ T cells constitute the major immune cell population; they interact with the peptide-major histocompatibility complex class I (MHC-I) molecules on antigen-presenting cells (APCs), differentiate to cytotoxic T lymphcytes (CTLs), and exhibit cytotoxicity against tumor cells (Iwahori 2020). Cytotoxic CD8^+^ T cells traffic to the tumor site to perform their cytotoxic function. Then, memory CD8^+^ T cells, as resident memory T (T_RM_) cells, either enter the tumor site or recirculate in the blood to perform their various functions as a central memory T (T_CM_) cells (Maimela et al. 2019).

The mechanisms by which CD4^+^ T cells influence anti-tumor immunity has been studied extensively. Activated CD4^+^ T cells secrete IL-2, which directly activates CD8^+^ CTLs expressing the IL-2 receptor α subunit (CD25) (Mackey et al. 1998; Bourgeois et al. 2002; Cheng et al. 2002; Tay et al. 2021). Additionally, CD4^+^ T cells indirectly mediate the activation and maturation of DCs, which activate CD8^+^ T cells either by cross-presenting tumor antigens or by producing effector cytokines such as IFN-γ and TNF-α. These cytokines have direct anti-tumor activity following activation and polarization of T cells into the T helper (T_H_) 1 phenotype (Ahrends and Borst 2018).

During interaction with DCs, activated CD4^+^ T cells can acquire neuropilin 1 (NRP1), a co-receptor that binds VEGF from DCs by an intercellular transfer mechanism (Bourbié-Vaudaine et al. 2006). The resulting NRP1-expressing T cells bind DC-secreted VEGFA and could potentially behave as VEGF-carrying cells, promoting angiogenesis (Bruno et al. 2014). Depletion of CD4^+^ T_H_1 cells decreases pericyte coverage and increases malformed tumor vessels in multiple mouse tumor models, whereas activation of CD4^+^ T cells improves vessel normalization (De Palma et al. 2017; Tian et al. 2017). T_H_1 cells also polarize M2-TAMs to M1-TAMs and induce DC maturation in the TME, which suppresses tumor angiogenesis (Heusinkveld et al. 2011; Motz and Coukos 2011). Furthermore, recent clinical evidence has highlighted the importance of CD4^+^ T cells in generating successful anti-tumor immunity. Single-cell RNA sequencing of T cells from colorectal cancer (CRC) patient biopsies indicates preferential enrichment of a T_H_1-like cell cluster of CD4^+^ T cells. These unique tumor-infiltrating CD4^+^ T cells express the transcription factor BHLHE40, the effector cytokine IFN-γ, and the CXC chemokine receptor (CXCR) 5, all of which are known to be expressed in T_H_1 cells (Zhang et al. 2018). Interestingly, the presence of T_H_1-polarised CD4^+^ T cells in the peripheral circulation has been found to be indicative of good prognosis in patients with non-small cell lung cancer (NSCLC) (Laheurte et al. 2019) or CRC (Ling et al. 2016).

CTLs secret large amounts of IFN-γ and TNF-α to kill infected or tumorigenic cells (Farhood et al. 2019; St Paul and Ohashi 2020). IFN-γ is one of the most potent effector cytokines secreted from both CD8^+^ and CD4^+^ T cells. Cytotoxic CD8^+^ T cells secrete IFN-γ, which suppresses tumor angiogenesis by inhibiting the proliferation of ECs and upregulating cytokine-encoding genes (e.g., CXCL9, CXCL10, and CXCL11). These cytokine-encoding genes stimulate the recruitment of pericytes, necessary for trafficking of immune cells across vessel walls (De Palma et al. 2017; Tian et al. 2017). Therefore, tumor-infiltrating T cells that secrete IFN-γ also contributes to vascular and immune remodeling.

### B cells

The important role of T cells in tumor immunosurveillance is well-established and has been extensively studied (Hashimoto et al. 2018; de Miguel and Calvo 2020; Yan et al. 2020; Ye et al. 2021). However, the function of B cells in this context is still poorly defined, although recent findings have suggested a role for B cells in the anti-tumor immune responses (Petitprez et al. 2020).

B cells mediate humoral immunity via their production of immunoglobulin molecules (antibodies). B cells undergo a diversification process during their development in the bone marrow. Naïve mature B cells that move into the periphery can be activated by antigen and become antibody-secreting plasma cells or memory B cells, which will respond more quickly to a second exposure to antigen (Packard and Cambier 2013). Activation of B cells occurs through different mechanisms, in either a T cell-dependent or T cell-independent manner (El Shikh et al. 2009).

The tumor-suppressing role of B cells in tumor progression and vascularization is complex and somewhat controversial. Tumor-infiltrating B cells may exert both pro- and anti-tumor responses depending on their phenotype and the antibodies they produce. Indeed, B cells in the TME have been described as being markers of both good and bad prognosis (Wouters and Nelson 2018). It has been reported that tumor-draining lymph node (TDLN) B cells have anti-tumor activities through direct killing of tumor cells via the Fas/FasL pathway. Activated TDLN B cells express Fas ligand, which is upregulated by co-culture with tumor cells. Effector B cells kill tumor cells directly in vitro in antigen-specific and Fas ligand-dependent manner (Tao et al. 2015). Activated TDLN B cells also mediated tumor regression after adoptive transfer into a murine pulmonary metastatic tumor model. These TDLN B cells produce cytotoxic antibodies such as IgM, IgG, and IgG2b, which bound specifically to tumor cells and led to specific tumor cells lysis in the presence of complement (Li et al. 2009b; Yuen et al. 2016). Although B cells can produce cytokines with CTL activity and serve as APCs, some studies indicate that regulatory B cells (Breg cells) are involved in pro-tumorigenic activities, through MDSCs, production of suppressive cytokines such as IL-10, IL-35 and TGF-β, and activation of immunosuppressive Treg cells (Schwartz et al. 2016; Sarvaria et al. 2017).

It has been reported that the interplay between B cells and ECs via the signal transducer and activator of transcription 3 (STAT3), an established and critical mediator of tumor angiogenesis (Yang et al. 2013). This arises from the potential to regulate VEGF expression (Gong et al. 2005). B cells with or without STAT3 have opposite effects on tumor growth and tumor angiogenesis in both B16 melanoma and Lewis lung cancer mouse models. Ex vivo angiogenesis assays show that B cell-mediated tumor angiogenesis is mainly dependent on the induction of pro-angiogenic gene expression, which requires STAT3 signaling in B cells. STAT3 is persistently activated in tumor-infiltrating B cells during tumor growth (Stockmann et al. 2014). Similarly, adoptive transfer of intrinsically activated STAT3-expressing B lymphocytes into implanted Rag1^−/−^ mice, lacking mature T or B cells, contributes to tumor growth and progression, whereas adding STAT3-deficient B cells to the TME results in reduced tumor development (Yang et al. 2013).

In contrast, Breg cells can kill macrophages, DCs and other immune cells during tumor development (Dasgupta et al. 2020). This phenomenon has been observed in several types of tumors, particularly in breast, ovarian, colorectal, cervical, and prostate cancers (Lindner et al. 2013). Recently, by bulk RNA sequencing, it was shown that B cells were different in the tumors of responders versus non-responders during immune checkpoint inhibitors (ICIs) treatment, implying that B-cells were predictive and potential therapeutic targets (Helmink et al. 2020). Future studies are needed to identify substances that can enhance the ability of B cells to facilitate anti-tumor immunity and the immunologic conditions that promote the pro-tumorigenic effects of B cells.

### Natural killer cells

In the early stages of tumor development, NK cells are part of the first line of defense against tumors and represent the cytotoxic compartment of the innate lymphoid cells (Barrow et al. 2019). NK cells are defined as CD3^−^ CD56^+^ cells in humans and are present in the peripheral blood, comprising approximately 5–15% of circulating lymphocytes (Guillerey et al. 2016).

The effector function of NK cells depends on the relative balance between the activating receptors [natural cytotoxicity receptors: NKp46, NKp44, NKp30, and NK group protein 2 family member D (NKG2) D] and the inhibitory receptors (killer inhibitory receptors, NKG2A, and killer cell lectin-like receptor subfamily G member 1) (Leibson 1997; Lanier 2001). Normal cells express MHC-I molecules, a ligand for inhibitory receptors on NK cells. However, cells experiencing various forms of stress, such as tumor cells, lose MHC-I expression while increasing expression of stress-associated molecules that act as ligands for NK activating receptors. As a result, the balance shifts toward the activation of NK cells and they release cytotoxic granules containing perforin and granzyme B to directly lyse tumor cells by death receptor-mediated pathways such as the Fas/FasL pathway. Furthermore, NK cells secrete pro-inflammatory cytokines and chemokines (such as IFN-γ, TNF-α, IL-6, granulocyte–macrophage colony-stimulating factor (GM-CSF) and CCL5) that might exert direct anti-tumor activity in addition to promoting innate and adaptive responses (Guillerey et al. 2016). NK cells can also produce type 2 humoral cytokines (e.g., IL-5 and IL-13) as well as immunoregulatory cytokines (e.g., IL-10 and TGF-β) (Lauwerys et al. 2000; Roda et al. 2006; Böttcher et al. 2018). Thus, NK cells are not only killers but also immunoregulatory cells that can positively or negatively influence anti-cancer responses by modulating the responses of DCs and T cells (Sungur and Murphy 2014). Another mechanism linked to the immunosurveillance of cancer by NK cells involves the elimination of senescent cells (Iannello et al. 2013).

Moreover, tumor-infiltrating NK cells operate within a hypoxic TME. As mentioned above, hypoxia has been extensively reported to modulate immune responses as well as drive angiogenesis (Schito and Semenza 2016). Hypoxia has been shown to impair NK cell cytotoxicity against multiple myeloma (MM). HIF-1α downregulates the expression of NK cell activating receptors such as NKp30, NKp44, and NKp46, NKG2D, granzyme B, and perforin (Sarkar et al. 2013). IL-2 has been utilized clinically after NK cell infusion (Shi et al. 2008; Sutlu and Alici 2009); similarly, pre-activation of NK cells by IL-2 eliminates the detrimental effects of hypoxia and increases expression of NKG2D. These activated NK cells can mediate cytotoxicity against MM, even under hypoxic conditions (Sarkar et al. 2013).

### Dendritic cells

Among all immune cells, DCs are the most potent professional APCs and are found in immunological organs such as the bone marrow, thymus, spleen, lymph nodes, and Peyer’s patches (Banchereau et al. 2000; Lanzavecchia and Sallusto 2001; Bonasio and von Andrian 2006). DCs play an important role in immunological processes by initiating, regulating, and maintaining immune responses (Fang et al. 2018). DCs arise from bone-marrow progenitors known as common myeloid progenitors and are classified into two alternative functional states: immature and mature cells. Immature DCs exhibit high levels of antigen uptake and processing and are unable to efficiently activate T cells. Conversely, mature DCs exhibit low levels of antigen uptake and increase activation of T cells (Dudek et al. 2013; Amigorena 2018).

Mature DCs are typically divided into two cell populations: the conventional DCs (cDCs) and plasmacytoid DCs (pDCs). Mature cDCs suppress tumor angiogenesis by secreting anti-angiogenic cytokines such as IL-12 and IL-18 as well as anti-angiogenic chemokines including chemokine CXC ligand (CXCL) 9, CXCL10, and CCL 21 (Trinchieri 2003; Curiel et al. 2004a; Piqueras et al. 2006). cDCs also are subdivided into two subtypes known as type 1 cDCs (cDC1) and type 2 cDCs (cDC2). cDC1 express the transcription factors basic leucine zipper transcriptional factor ATF-like 3 (BATF3) and interferon regulatory factor (IRF) 8. cDC1 specialize in cross-presentation of exogenous antigen to activate CD8^+^ T cell-mediated immunity (Sichien et al. 2017). The BATF3 transcription factor plays an important role in anti-tumor immune responses and impacts cancer immunotherapies, such as immune-checkpoint blockade and adoptive transfer T cell therapy (Spranger et al. 2017). BATF3-dependent cDC1 are critical for regulating the infiltration of CD8^+^ T cells into tumor tissue (Sánchez-Paulete et al. 2016). cDC1 are the major source of CD8^+^ T cell chemoattractants CXCL9 and CXCL10; these chemokines drive T cell recruitment and anti-tumor immunity (Spranger et al. 2017; de Mingo Pulido et al. 2018) as well as suppress angiogenesis by secretion of anti-angiogenic factor such as IL-12 and IL-18 (Curiel et al. 2004a; Piqueras et al. 2006). pDCs secrete IFN-α, which inhibits proliferation and motility of ECs and increases expression of anti-angiogenic cytokines and chemokines through Toll-like receptor (TLR) 7 or signaling (Indraccolo et al. 2002; Asselin-Paturel and Trinchieri 2005; Kawai and Akira 2011). Within the TME, cytokines produced by DCs may induce the activation and proliferation of Treg cells (Li et al. 2021). A novel subset of tolerogenic DCs can also promote the differentiation of Treg cells through producing high levels of IL-10 (Gregori et al. 2010). DCs can secrete CCL22 that promotes interactions between DCs and Treg cells via binding to its receptor CCR4. The recruitment of Treg cells into the tumors cause immune suppression and downregulation of co-stimulatory molecules on DCs, causing CTLs dysfunction (Curiel et al. 2004b; Bauer et al. 2014; Rapp et al. 2019).

### Tumor-associated macrophages

Macrophages are specialized phagocytic cells that not only present antigen but also clear pathogens and cell debris. Macrophages can be categorized into classic M1 macrophages (pro-inflammatory polarization) and alternative M2 macrophages (anti-inflammatory polarization) depending on the signals from the surrounding microenvironment. M1 macrophages are capable of pro-inflammatory responses and inhibit the proliferation of malignant cells by secreting pro-inflammatory cytokines such as TNF-α, IL-1, IL-6, IL-12, IL-18 and IL-23 (Magdalena Klink 2016; Mills et al. 2016). These cytokines attract additional unpolarized macrophages to the M1 state. M1 macrophages produce nitric oxide (NO) or reactive oxygen intermediates to increase their pathogen-killing ability. M2 macrophages are capable of anti-inflammatory responses by enhancing the secretion of IL-10 and reducing the secretion of IL-12 and IL-23 (Arora et al. 2018). They are involved in anti-inflammatory effects and promote tissue repair and wound healing (Kim and Nair 2019).

TAMs generally represent the major component of myeloid cells in the TME, affecting tumor initiation, progression, angiogenesis, and metastasis (Mantovani et al. 2017). A high-level infiltration of TAMs is associated with poor prognosis in various types of cancer, with the exception of CRC (Hasita et al. 2010; Zhang et al. 2012, 2019; Jung et al. 2015; Ruffell and Coussens 2015; Wan et al. 2015; Yeung et al. 2015; Zhou et al. 2016; Kitano et al. 2018). In the early stages of tumorigenesis, TAMs, and in particular M1 macrophages, suppress sprouting angiogenesis and induce vessel maturation by secreting anti-angiogenic cytokines (e.g., IL-12 and TNF-α) and activate an anti-tumor immune response (Qian and Pollard 2010; Chen and Bonaldo 2013). Accordingly, immunotherapy with IL-12 not only reduces microvessel density but also enhances M1 macrophage polarization in tumors (Watkins et al. 2007; Chen and Bonaldo 2013). However, TAMs can be transformed from the M1 to M2 phenotype to promote tumor angiogenesis by producing pro-angiogenic growth factors (e.g., VEGF, EGF, FGF family, and PDGF-β), angiogenic CXC chemokines (e.g., CXCL8/IL-8 and CXCL12, also known as SDF-1), and angiogenesis-associated factors (e.g., TGF-β, thymidine phosphorylase) (Muller et al. 2001; Lewis and Pollard 2006; Kim et al. 2012; Hughes et al. 2015).

Overall, TAMs are a double-edged sword as they can function as both “tumor promoters” and “tumor suppressors” in the TME as they promote tumor angiogenesis yet also act as central drivers of the immunosuppressive TME by producing different growth factors, chemokines, and angiogenesis-associated factors (Chen and Bonaldo 2013; Rivera and Bergers 2015). Their dual function provides a unique therapeutic opportunity to target macrophages via cancer immunotherapy. In fact, eliminating macrophages from tumor sites, through genetic or therapeutic means, has been shown to reduce tumor progression in breast cancer (Laoui et al. 2011).

## Tumor-promoting immune cells

Besides the tumor-antagonizing immune cells, there are a plenty of tumor-promoting immune cells mainly consisting of M2-tumor associated macrophages, Tregs cells MDSCs and Bregs cells.

### Regulatory T cells

Treg cells are a prominent immunosuppressive subpopulation of CD4^+^ T cells that inhibit immune responses, thereby maintaining homeostasis and limiting immune activation (Dadey et al. 2020). Several mechanisms by which Treg cells suppress immune function have been reported: secretion of immunoregulatory cytokines such as TGF-β, IL-10, and IL-35 (Romano et al. 2019; Sullivan et al. 2020); cytolysis of effector cells via secretion of granzyme and perforin (Arce-Sillas et al. 2016); and metabolic interruption by cyclic adenosine monophosphate (cAMP)-mediated immunosuppression. Treg cells generate and accumulate high levels of cAMP, which they then transfer to target cells using intercellular communication through gap junctions (Rueda et al. 2016).

In addition, Treg cells express the transcription factor, forkhead box p3 (FOXP3). *FOXP3* is critical for their differentiation and function, including the secretion of suppressive cytokines (e.g., TGF-β, IL-10, and IL-35) and the expression of inhibitory surface molecules such as cytotoxic T cell-associated antigen-4 (CTLA-4) and glucocorticoid-induced TNF receptor family-related gene/protein (GITR). These inhibitory surface molecules repress the production of IL-2, IFN-γ, and IL-4 (Fontenot et al. 2003b; Hori et al. 2003; Josefowicz et al. 2012). Deficiency of *Foxp3* in mice impairs the development of Treg cells and rapidly causes fatal autoimmunity and lymphoproliferative disease (Fontenot et al. 2003a; Khattri et al. 2003). In humans, *FOXP3* mutation results in IPEX syndrome (immune dysregulation, polyendocrinopathy, enteropathy, X-linked), a severe autoimmune disease (Bennett et al. 2001).

Treg cells are found at high frequencies in a variety of tumors such as breast (Liyanage et al. 2002), lung (Wolf et al. 2003), liver (Ormandy et al. 2005), gastrointestinal tract (Ichihara et al. 2003; Sasada et al. 2003), pancreas (Hiraoka et al. 2006) and ovary (Sato et al. 2005). Their high frequency among CD4^+^ T cells in tumor-infiltrating lymphocytes or a high ratio of FOXP3^+^ Treg cells to CD8^+^ T cells is associated with worse prognosis, especially in patients with breast (Bates et al. 2006), gastric (Sasada et al. 2003), and ovarian cancer (Sato et al. 2005). Tumor hypoxia results in expression of CCL28, which promotes the recruitment of CD4^+^CD25^+^FOXP3^+^ Treg cells by binding to the corresponding receptor, CCR10, on Treg cells. Forced expression of CCL28 in mouse ovarian cancer cells causes robust Treg cells accumulation, but also results in increased VEGF levels and significantly increases blood vessel development, which is associated with rapid tumor angiogenesis (Motz and Coukos 2011; Facciabene et al. 2012). Although this pro-angiogenic effect could be indirect, the depletion of Treg cells in ovarian tumor-bearing-mice correlated with a strong reduction of the VEGF at the tumor site, suggesting a role of Treg cells in promoting tumor angiogenesis in ovarian cancer (Facciabene et al. 2011). Therefore, the presence of Treg cells in the TME plays a critical role in both the anti-tumor immune response and tumor angiogenesis.

### Myeloid-derived suppressor cells

MDSCs are a heterogenous population of immature myeloid cells that play a critical role in tumor-associated immune suppression (Gabrilovich and Nagaraj 2009; Molon et al. 2011; Kumar et al. 2016; Shipp et al. 2016). About 10 years ago, two major subsets of MDSCs were identified based on their phenotypes and morphological features: granulocytic/polymorphonuclear MDSCs (G-MDSCs/PMN-MDSCs) and monocytic MDSCs (M-MDSCs). G-MDSCs are phenotypically and morphologically similar to neutrophils, whereas M-MDSCs resemble monocytes (Kumar et al. 2016). Both types of MDSCs have inhibitory effects in mouse tumor models and several human cancers (Luker et al. 2020).

One of the main features of MDSCs is their ability to suppress the immune cell function. MDSCs mediate immune suppression through the expression of arginase 1 (ARG1), inducible nitric oxide synthase (iNOS; Youn et al. 2008; Dolcetti et al. 2010), TGF-β (Huang et al. 2006; Li et al. 2009a), IL-10 (Stockmann et al. 2014; Albini et al. 2018), cyclooxygenase 2 (COX2; Fujita et al. 2011; Mao et al. 2013), and indoleamine 2,3-dioxygenase (IDO) chelating cysteine (Srivastava et al. 2010) among others. The inhibition of T cell proliferation is most important in evaluating the activity of MDSCs (Gao et al. 2020).

In addition to the establishment of an immunosuppressive TME, MDSCs could promote tumor progression by non-immunological mechanisms (Safarzadeh et al. 2018). MDSCs promote de novo angiogenesis via different mechanisms. Mainly, MDSCs promote neo-angiogenesis by secreting growth factors like VEGF, bFGF, Bv8, and PDGF. Additionally, they remodel the extracellular environment via MMP production. Moreover, they are capable of reprogramming other cells to a tumor-promoting phenotype, which in turn can promote angiogenesis via the secretion of proangiogenic factors like VEGF (Vetsika et al. 2019). MDSCs enhance tumor angiogenesis by increasing expression of IL-10 and decreasing expression of IL-12 (Sinha et al. 2007; Murdoch et al. 2008; Stockmann et al. 2014; Albini et al. 2018). In particular,  MDSCs produce large amounts of MMPs, especially MMP9, which degrade ECM and basement membrane, and enable tumor to enter the blood stream for migration into the metastatic site (Baniyash 2016). MDSCs promote angiogenesis by secreting elevated levels of VEGF and bFGF (Shen et al. 2014).

In the TME, MDSCs deprive T cells of essential amino acids via the STAT/MyD88 signaling pathway to up-regulate metabolic enzymes (e.g., ARG1 and iNOS) (Gabrilovich et al. 2012; Melero-Jerez et al. 2016). MDSCs also increase the uptake of cationic amino acid transporter 2B (CAT2B) and glutamate/cysteine antiporter solute carrier family 7 member 11 (SLC7A11), thereby increasing the consumption and intracellular degradation of arginine and cysteine (Groth et al. 2019). In fact, Arg is metabolized by iNOS, generating citrulline and NO to suppress the activation of T cells, decrease expression of MHC-II molecules on APCs, and further induce differentiation of Treg cells (Lee et al. 2016) and polarization of T_H_17 cells (Nagaraj et al. 2013; Wen et al. 2016). In addition, MDSCs infiltrating the spleen and tumor decrease the antigen-specific immune response of T cells, both in mice and patients with head and neck cancer (Young and Lathers 1999; Srivastava et al. 2010). Although the MDSCs are well-known regulators of Treg cells in different types of cancer, recent findings also demonstrate that Treg-derived TGF-β is a crucial controller of the proliferation and function of MDSCs (Lee et al. 2016). However, more research is needed to better dissect the relationship between MDSCs and Tregs in the TME.

## Vascular normalization and immune responses

Administration of bevacitumab (Avastin®), an anti-VEGF antibody approved by the FDA, did not significantly improve the prognosis of colorectal and glioblastoma patients (Hubbard and Rothlein 2000). This is likely due to the increased expression of various angiogenic factors induced by the re-appearance of hypoxia caused by the inhibition of angiogenesis. Upregulation Ang-2/Tie2 signaling pathway may contribute to the resistance of VEGF inhibition therapy (Chae et al. 2010). However, co-administration of bevacitumab with other angiogenesis pathway blockers or anti-cancer drugs resulted in clinically meaningful increases in overall survival in patients with CRC, cervical cancer, or NSCLC (Sandler et al. 2006; Kindler et al. 2012; Tewari et al. 2014).

A pioneering scientist, Rakesh Jain, proposes that the TME can be changed by correcting the abnormalities in cancer blood vessels (Jain 2014). His group first attempted to normalize cancer blood vessels through an effort to correct the imbalance between pro-angiogenic and anti-angiogenic factors because the abnormality in the tumor blood vessels results from this imbalance. This strategy is termed “vascular normalization” (Jain 2001). The strategy in “vascular normalization” is to administer the appropriate dose of angiogenesis inhibitors over a short time frame, with or without co-administration with other anti-cancer drugs during the short period (days to weeks) called the “normalization window” (Jain 2005). The dosage of anti-angiogenic therapy is an important concern in order to achieve vascular normalization. High-doses of anti-VEGF agents have a short normalization window due to excessive vascular pruning, but lead to hypoxia and acidification of the TME. High-doses of anti-VEGF agents promote infiltration of MDSCs, Treg cells, and M2-like macrophages; M2-like macrophages are pro-tumoral immune cells that induce not only hypoxia but also accumulation of ECM, causing immunosuppression. On the other hand, it was observed that if anti-angiogenic agents were used at quarter of the dose that resulted in the above side effects, vascular pruning or anti-angiogenic effects were induced, leading to continuous vascular normalization in animals. Vascular normalization serves as an effective penetration channel for immune cells. If tumor oxygen supply is increased by the enhanced perfusion, the anti-cancer activity of the infiltrating immune cells is increased (Jain 2014). Mechanistically, anti-VEGF-A/Ang-2 treatment improved perfusion and reduced leakiness of the remaining tumor vasculature in a phenotype that was associated with increased perivascular accumulation and intra-tumoral infiltration of CD8^+^ T cells (Kashyap et al. 2020).

## Conclusion

Tumors receive blood supply via new blood vessel formation, such as angiogenesis induced by the hypoxic TME. A lot of molecules have been identified to play a critical role in new blood vessel formation, but VEGF and angiopoietin signaling are the most studied and stimulated by hypoxia. A highly active angiogenic process induces structurally and functionally abnormal vasculature, limiting blood perfusion and infiltration of immune reactive cells for killing tumor cells. Crosstalk between the abnormal tumor vasculature and various immune cells determine the immune reactive or immune suppressive states in TME. Understanding this crosstalk creates the strategies on the immune activation in TME to cure various cancers. One of the outstanding approaches is the normalization of tumor vasculature by the anti-angiogenic reagents within a short period with the chemotherapy and radiotherapy. In the future, we will discuss and review about the combination therapy of chemo- or radio-therapy with anti-angiogenic treatment and its clinical applications.
